# Fatal Cerebral Edema in a Young Adult with Diabetic Ketoacidosis: Blame the Bicarbonate?

**DOI:** 10.1155/2020/5917459

**Published:** 2020-04-30

**Authors:** Sameera Natarajan, Radhika Kulkarni, Apoorva Tangri

**Affiliations:** ^1^Advocate Illinois Masonic Medical Center, 836 W Wellington Ave., Chicago, IL 60657, USA; ^2^University of Connecticut: UConn Health, 263 Farmington Ave., Farmington, CT 06030, USA; ^3^Rush University Medical Center, 1750 W Harrison St., Jelke Building South, Suite 444, Chicago, IL 60612, USA

## Abstract

Cerebral edema is a devastating complication of DKA which is extremely rare in adults but is the leading cause of diabetes-related death in the pediatric population. Newly diagnosed diabetes, younger age, first episode of DKA, severity of DKA at presentation, and administration of bicarbonate are predictive of cerebral edema in DKA. We present a case of a young adult with DKA as the presenting symptom of diabetes, whose clinical course was complicated by renal failure, refractory shock, and cerebral edema. This case addresses the controversy surrounding bicarbonate therapy in DKA and its possible role in the development of a rare fatal complication of DKA.

## 1. Introduction

Diabetic ketoacidosis (DKA) is a serious acute complication of diabetes mellitus. The combination of insulin deficiency and an increase in counterregulatory hormones results in severe metabolic derangements. Cerebral edema (CE) is a devastating complication of DKA that is well-described in the pediatric population but exceptionally rare in adults. We present a case of a morbidly obese young adult with no known history of diabetes who presented with DKA and developed multiorgan failure and cerebral edema.

## 2. Case Presentation

A young Caucasian male in the third decade of life presented to the emergency room with confusion and tachypnea. He had a 2-week history of generalized weakness and a 2-day history of nausea, vomiting, diarrhea, polyuria, and polydipsia. Apart from morbid obesity (weight 183 kg, BMI 60.4 kg/m^2^), he had no known chronic medical problems. He was on no medications and denied exposure to drugs, alcohol, or hazardous chemicals. He had multiple first-degree relatives with diabetes mellitus, but it is unknown if they had type 1 or type 2 diabetes mellitus. On initial examination, he was afebrile, tachypneic, tachycardic, and hypertensive, with normal oxygen saturation on room air. He was alert and responsive (GCS 13) but noted to be dehydrated and in severe respiratory distress. His cardiopulmonary examination was unremarkable except for a regular pulse rate of 100/min. His initial random blood glucose of 694 mg/dL, bicarbonate of 5 mmol/L, arterial pH of 6.86 and calculated anion gap of 43 satisfied all the diagnostic criteria for DKA. He was oliguric with serum creatinine 1.37 mg/dL, potassium 3.5 mmol/L, and serum sodium (corrected for hyperglycemia) 138 mmol/L. His white blood cell count was 19,500/mm^3^. The arterial blood gas panel was consistent with primary metabolic acidosis with appropriate respiratory response, which was attributable to ketoacidosis and lactic acidosis (venous lactic acid was 4.3 mmol/L). Urinalysis revealed glucosuria, proteinuria (100 mg/dL), and ketonuria and was not suggestive of infection. Chest X-ray showed crowding of bronchovascular structures (likely related to suboptimal inspiration) and a streaky left retrocardiac opacity suggestive of atelectasis. He was given intravenous fluid boluses (3 liters normal saline), empiric antibiotic coverage with piperacillin-tazobactam, intravenous potassium chloride (40 mEq), and intravenous calcium gluconate. Intravenous insulin infusion was started at the rate of 8 units/hour and thereafter titrated per the hospital's diabetic ketoacidosis protocol. Concurrently, another 80 mEq of KCl was given intravenously. Repeat serum potassium was 3.0 mmol/L, and the insulin infusion was paused per protocol to prevent further hypokalemia. Despite receiving an additional 30 mmol potassium phosphate intravenously and 140 mEq potassium chloride enterally, the hypokalemia worsened (serum potassium 2.7 mmol/L). Due to lack of improvement in severe acidosis, he was given 100 mEq and then another 150 mEq of sodium bicarbonate intravenously, and an infusion of 100 mEq sodium bicarbonate in 0.45% sodium chloride was begun at the rate of 250 mL/hr. He became hypothermic (bladder temperature 35.0 degrees Celsius) and hypotensive (96/27 mmHg) and was started on vasopressor support with norepinephrine. He remained severely acidotic (arterial pH 6.99) with worsening hypoxemia and hypercarbia. He was intubated and mechanically ventilated using assist control-volume control mode, with a tidal volume of 600 mL and a minimum set respiratory rate of 25/min. A chest X-ray revealed interval increase of interstitial markings, a new left retrocardiac opacity, and patchy perihilar and bibasilar opacities, suggesting pulmonary congestion and possible developing pneumonia. He went into anuric renal failure, and continuous venovenous hemofiltration was initiated but could not be maintained due to profound hypotension and subsequent clotting of the circuit. At this stage, the patient was on norepinephrine, vasopressin, and phenylephrine infusions to maintain his mean arterial pressure over 65 mmHg. It was then noted that he was not responsive to painful stimuli despite not being on any sedation for several hours. Pupils were unequal and nonreactive to light. An emergent noncontrast computed tomography of the head revealed diffuse cerebral edema with transtentorial and cerebellar tonsillar herniation, for which a dose of mannitol was administered. Due to his poor prognosis, the next of kin elected to withdraw life-sustaining measures, and the patient passed away. Posthumously, laboratory results came back with HbA1C of 12.9%, and antibodies to islet cell, glutamic acid decarboxylase, and insulin were negative, suggesting the patient had underlying type 2 diabetes mellitus.

## 3. Discussion

Diabetic ketoacidosis (DKA) is a serious and potentially life-threatening complication of diabetes mellitus. Although typically associated with insulin-deficient states like type 1 diabetes, it may also occur in type 2 diabetes in conditions of extreme physiologic stress or in a variant of type 2 diabetes called ketosis-prone diabetes [1]. DKA is characterized by hyperglycemia (although blood sugars may be normal), ketosis, and high anion gap metabolic acidosis [1, 2]. DKA can occur in persons of all ages, but nearly 65% of cases occur in persons 50 years of age and below [3]. In the United States, hospitalizations for DKA are on the rise, while mortality rates have steadily downtrended, with in-hospital case fatality rates of <1% reported in recent years [4]. The cause of death in DKA is usually the inciting event [5]. Volume depletion is a hallmark of the disease process and is primarily attributed to glucose-driven osmotic diuresis [6]. The management of DKA is challenging, requiring repletion of intravascular volume, titration of insulin, and correction of acid-base and electrolyte imbalances. Neurologic manifestations such as obtundation, seizures, and coma may be seen in DKA when serum osmolality is severely increased [6].

Cerebral edema (CE) is a devastating complication of DKA. It is well-described in the pediatric population—it occurs in 0.2-1% of children with DKA and is the leading cause of diabetes-related death in this age group [7–9]. The rarity of CE in adults with DKA is illustrated by the fact that there are very few reported cases in this age group. From our review, we identified only a handful of case reports in the literature in the past two decades describing this rare complication of DKA in adults [10–15]. About half of the patients died, and the ones who survived had no residual neurological deficits. Of interest, most had no known history of diabetes. The age specificity of this disease remains unexplained, but irrespective of age, it is associated with significant morbidity and mortality—21-25% die and 15-26% of survivors are left with neurological impairments [16]. A review of 69 instances of intracerebral complications in DKA, of which most of the subjects were children under the age of five years and newly diagnosed diabetics, found that half exhibited clinical signs and symptoms of neurological deterioration prior to respiratory arrest. Despite early recognition and intervention in this group, 50% had severe or fatal brain damage [17]. This sheds light on the fact that most studies do not account for subclinical CE which is far more prevalent [18]. Factors associated with development of CE include newly diagnosed diabetes, younger age, first episode of DKA, severity of DKA at presentation, administration of bicarbonate, and sluggish improvement of hyponatremia with treatment [8].

Although the precise pathogenesis of CE in DKA remains poorly understood, a widely accepted theory blames aggressive intravenous rehydration, use of hypotonic fluids, rapid correction of hyperglycemia, and fluctuating oncotic pressures, all together causing an osmotic shift of fluid into the intracellular space resulting in cell swelling [19, 20]. On this basis, treatment protocols adopted a strategy of slower intravenous hydration with isotonic fluids [5]. The patient discussed in this report received 5.5 L of IV fluids in the first 12 hours after presentation to the hospital. It is conceivable that the studies are biased to the fact that patients who developed cerebral edema were more likely to have severe DKA and therefore be more dehydrated and thus appropriately receive larger amounts of fluids. A 13-center randomized controlled trial of 1389 episodes of pediatric DKA randomly assigned children to one of four treatment groups in a 4 × 4 factorial design—0.9% or 0.45% sodium chloride content and fast or slow rate of administration. There were no significant differences observed in the rate of clinically apparent brain injury during treatment or in neurocognitive function after recovery, among patients in the four treatment groups [21]. Thus, neither the rate of administration nor the sodium chloride content of intravenous fluids significantly influenced neurological outcomes, underscoring a lack of causal association between rapid fluid administration and DKA-related brain injury.

Another school of thought implicates the effects of cerebral reperfusion, neuroinflammation, and disruption of the blood-brain barrier in the development of CE in DKA [8, 19]. Some studies describe that CE in DKA is related to cerebral ischemia [8]. Both hypocapnia (which causes cerebral vasoconstriction) and intravascular volume depletion would be expected to cause cerebral hypoperfusion [8].

In general, severe acidemia causes impaired myocardial contractility and decreased peripheral vascular resistance [22]. However, the addition of bicarbonate therapy to correct metabolic acidosis, particularly in DKA, has been shown to be unhelpful and in some cases even harmful [23]. Except in severely acidemic patients, it is believed that insulin therapy by inhibiting lipolysis will correct the acidosis without use of bicarbonate [6, 9]. Bicarbonate administration in metabolic acidosis is associated with adverse effects such as hypokalemia, hypercapnia, ionized hypocalcemia, and the aforementioned CE [23]. Theoretically, correction of acidosis also increases hemoglobin affinity for oxygen, thus decreasing tissue oxygenation [24]. Okuda et al. demonstrated that in DKA, bicarbonate therapy markedly increased hepatic production of acetoacetate and *β*-hydroxybutyrate levels [25]. This shows that alkali loading, although reversing the acidemia, is counterproductive in reversing the increased ketogenesis in DKA. A review of adults with DKA with initial pH < 7.0 showed that intravenous bicarbonate therapy does not significantly hasten resolution of acidosis or decrease the time-to-hospital discharge [26]. Additionally, patients who received intravenous bicarbonate were shown to have significantly higher insulin and fluid requirements in the first 24 hours compared to those who did not. A randomized prospective study of twenty-one patients failed to show a difference in recovery outcome variables with bicarbonate administration in adults with severe DKA with arterial pH 6.9 to 7.14 [27].

Total body potassium is low upon initial presentation in DKA, due to osmotic diuresis, poor oral intake, and gastrointestinal losses. However, the serum potassium level is often normal or high, due to the extracellular shift of potassium in the setting of insulin deficiency and acidosis [28, 29]. A small percentage of patients (5.6% in one prospective study) may present with hypokalemia [30]. This is likely a sign of profoundly depleted total body potassium due to a prolonged untreated hyperosmolar state and only in rare cases secondary to preexisting renal tubular acidosis, malabsorption, or malnutrition [28]. In these patients, potassium repletion until serum potassium concentration is >3.3 mEq/L is recommended prior to starting insulin [29].

Current guidelines from the American Diabetes Association recommend that adults with pH lower than 6.9 should receive an isotonic solution composed of 100 mmol sodium bicarbonate in 400 mL sterile water with 20 mEq KCl, given at a rate of 200 mL per hour for two hours until the venous pH > 7.0. If the pH is still lower than 7.0 after this is infused, they recommend repeating this infusion every two hours until pH reaches 7.0 [29]. The existing literature includes very few subjects with a pH of <6.9 on presentation, highlighting the need for additional research in severe acidemia. A definitive study on the risk-benefit ratio of bicarbonate in DKA requires a larger number of patients to provide enough power for conclusive results. Until then, it seems prudent for patients with pH < 6.9 to be given bicarbonate therapy.
